# The Validation of an Innovative Method for 3D Capture and Analysis of the Nasolabial Region in Cleft Cases

**DOI:** 10.1177/1055665620946987

**Published:** 2020-08-12

**Authors:** Ashraf Ayoub, Adil Khan, Ali Aldhanhani, Hashim Alnaser, Kurt Naudi, Xiangyang Ju, Toby Gillgrass, Peter Mossey

**Affiliations:** 1 Oral & Maxillofacial Surgery, Glasgow University Dental Hospital and School, Glasgow, United Kingdom; 2 Oral & Maxillofacial Surgery, University of Glasgow Dental Hospital and School, Glasgow, United Kingdom; 3 Oral Surgery, University of Glasgow Dental Hospital and School, Glasgow, United Kingdom; 4 Image Processing, Medical Devices Unit, NHS Greater Glasgow and Clyde, Glasgow, United Kingdom; 5 Orthodontics, Glasgow Dental Hospital and School, University of Glasgow, Glasgow, United Kingdom; 6 Craniofacial development, 3042 Dundee University, Dundee, United Kingdom

**Keywords:** cleft, intraoral scanner, 3D, imaging, scar, asymmetry

## Abstract

**Objective:**

To validate a newly developed method for capturing 3-dimensional (3D) images of the nasolabial region for assessing upper lip scarring and asymmetry in surgically managed unilateral cleft lip and palate (UCLP) cases.

**Design:**

Validation study, single cohort.

**Materials and Methods:**

Eighteen surgically managed UCLP cases were recruited, the nasolabial region of each face was scanned using an intraoral scanner (IOS) to produce 3D images. The images were manually segmented to allow the calculation of surface area of the scar and upper lip asymmetry. Five professionals and 5 lay assessors subjectively evaluated the same images and graded the upper lip scarring and asymmetry at 2 separate occasions. The relationship between the subjective and objective assessments was evaluated.

**Results:**

Moderate correlation was found between subjective and objective evaluations of the upper lip scarring and total asymmetry. The captured 3D images were of good quality for the objective measurements of lip asymmetry and residual scarring. Moderate to strong correlations were detected between the 2 panels (T ranging between 0.5 and 0.9) with no significant difference (*P* > .05) in the mean score of the subjectively evaluated parameters.

**Conclusion:**

The IOS is a useful tool for the capture of the nasolabial morphology. The captured 3D images are a reliable source for measuring lip asymmetry and scar surface area. The method has sufficient validity for routine clinical use and for objective outcome measures of the surgical repair of cleft lip.

## Introduction

The evaluation of facial appearance of the unilateral cleft lip and palate (UCLP) has been considered both qualitatively and quantitatively (Al-Omari et al., 2015, Thierens et al., 2018). This included the direct clinical anthropometry, 2-dimensional (2D), 3-dimensional (3D) imaging, and clinical videographs (Thierens et al., 2018). Direct clinical measurements are time-consuming, require the cooperation of the patient, pose risks of injury, and only identify the most obvious disproportions of the face (Ferrario et al., 2003). The objective assessment of 2D photographs of UCLP cases has satisfactory reproducibility (Asher-McDaid et al., 1991). However, the method has clear deficiencies due to the posing discrepancy, the magnification error and the limited visual perspective offered by a 2D image which is not reflective of observers view in real life lack of capturing the third dimension. Three-dimensional imaging methods provide a comprehensive recording of the facial morphology that lends itself to both objective and subjective assessment (Stebel et al., 2016; Thierens et al., 2018).

An ideal imaging technology of the facial region should fulfill the following criteria: fast capturing time of the face, accuracy within 1 mm, simple acquisition of the 3D morphology, cost-effectiveness and minimal exposure of patients to harmful radiation (Ayoub et al., 2003). Various 3D imaging methods were used for capturing the orofacial morphology for the quantitative analysis and characterization of the cleft-related facial deformity, which include computed tomography scans (CT), cone beam CTs (CBCT), magnetic resonance imaging (MRI), stereophotogrammetry, structured light surface scanning, and laser surface scanning technologies (Brons et al., 2012; Kuijpers et al., 2014; Awarun et al., 2019). Computed tomography and CBCT scans provide images of both the hard and soft tissues; however, the risk of exposure to ionizing radiation renders them inappropriate for the routine capturing and longitudinal evaluation of facial morphology. Metallic objects such as dental restorations and orthodontic braces produce streak artifacts that distort facial images of the CT and MRI scans (Ayoub et al., 1998). Moreover, the soft tissue resolution is poor and the captured images lack the natural photorealistic appearance of the face. The relatively prolonged acquisition time is the main disadvantage of the structured light and laser technologies, which limit their application in infants and young children (Lane and Harrell, 2008). Additionally, laser light can pose a hazard to the retina of the eye. Stereophotogrammetry offers safety, speedy acquisition within one millisecond, and accuracy of 0.5 mm in recording facial morphology. These advantages over other imaging technologies allowed the capture of the face of infants and young children (Ayoub et al., 2003). Intraoral scanners (IOSs) provide an alternative method for capturing 3D images (Richert et al., 2017; Mangano et al., 2017). A recent systematic review reported that IOS are accurate within 140 µm and 1330 µm (Bohner et al., 2019). Intraoral scanners are mainly utilized for capturing the dental occlusion as an aid to diagnosis and planning in prosthetic rehabilitation, restorative, and orthodontic treatments. Recent studies have also reported extraoral applications for IOS, namely fabrication of maxillofacial prostheses. Liu et al. (2019) reported on the fabrication of orbital prostheses using the images generated by IOS. This method entails capturing the healthy eye, then creating a mirror copy of the image to reconstruct the defect on the contralateral side. They claimed that the IOS accuracy was clinically sufficient and can be applied for image capture and planning to restore maxillofacial defects. Similarly, Ballo et al. (2019) reported a technique for prosthetic auricular rehabilitation by direct digital scanning of the unaffected ear using IOS. The authors argued IOS could produce a better 3D model of the ear than the other imaging technologies because of the high level of accuracy in recording the complex anatomy of this region with multiple anatomical undercuts. They concluded that the application of IOS for maxillofacial imaging achieved excellent outcome, eliminated the need for manual sculpting, and is more time-efficient in comparison to the conventional methods. The IOS has not yet been applied to the recording of facial morphology or the nasolabial region in CLP cases.

Cleft-related facial deformity has been quantified using a wide variety of objective measurements including simple linear, angular, and volumetric measurements to more complex asymmetry analyses including distance mapping following the superimposition of original and mirrored scans. Facial shape analysis was achieved using Generalized Procrustes analysis, Bookstein analysis, principal component analysis, and/or canonical variate analysis (Hallac et al., 2017, Al Rudainy et al., 2019). Recently, the dynamic of lip movements in surgically managed UCLP cases has been investigated using 4D imaging system (Gattani et al, 2020).

On the other hand, subjective assessments of cleft-related facial disharmony may still assessed using visual analog scales and Likert-type ordinal scales. Subjective assessment lacks reproducibility and the limited agreement among assessors is well-documented (Mosmuller et al., 2017). They assessed the reliability of various objective and subjective assessments of 2D and 3D images of the nasolabial region of surgically managed UCLP cases. The nasolabial asymmetry was measured by means of distance mapping, while a lay panel composed of 8 judges evaluated the nasolabial appearance. The subjective assessment of the 3D images was the least reliable. The study highlighted the importance of the objective measurement of the nasolabial morphology and further research on the assessment techniques that are clinically valid.

The main aim of the present study was to assess the reliability and validity of IOS for recording 3D images of the nasolabial region to quantify residual dysmorphology in surgically managed UCLP cases.

## Material and Methods

Written consent was obtained from each patient/parent prior to scanning the nasolabial region, and all patients/parents agreed that the images could be included for research purposes. The photographs were anonymized.

**Figure 1.**
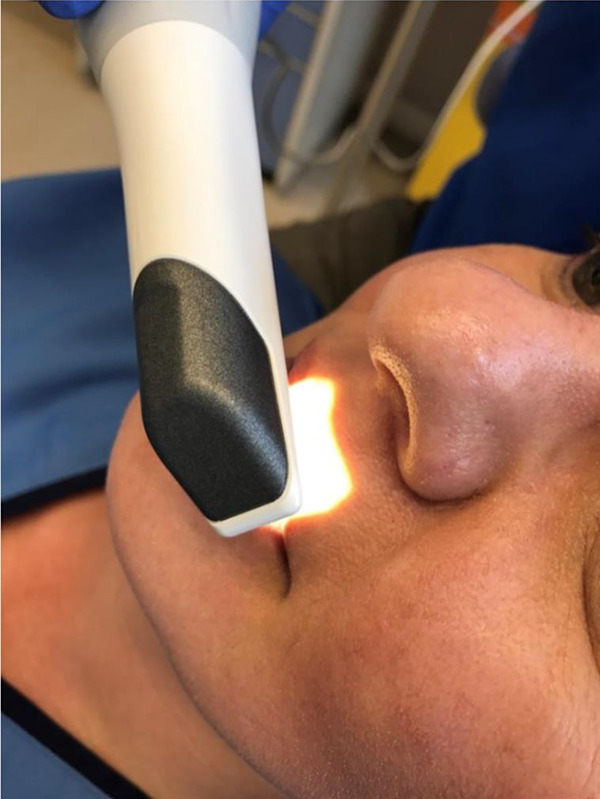
The use of the intraoral camera to record the 3-dimensional (3D) morphology of the nasolabial region.

**Figure 2.**
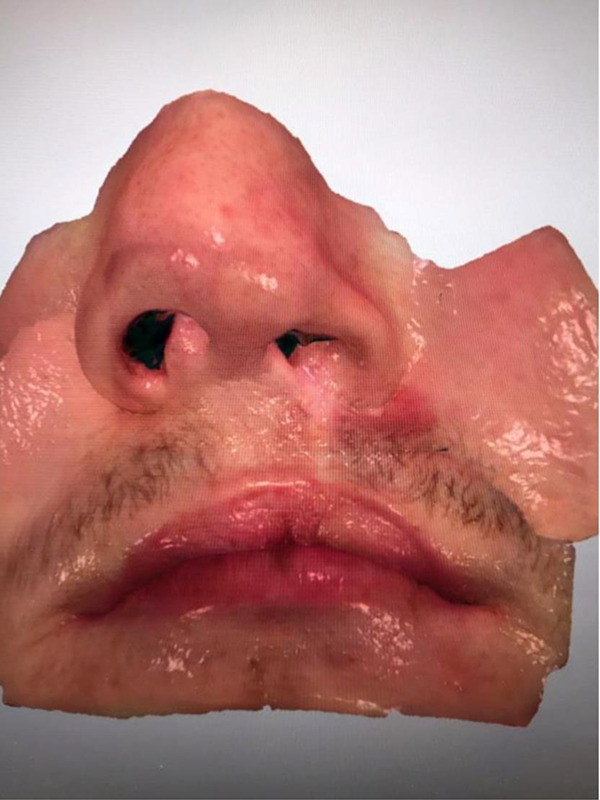
An example of the 3-dimensional (3D) image captured by the intraoral camera.

**Figure 3.**
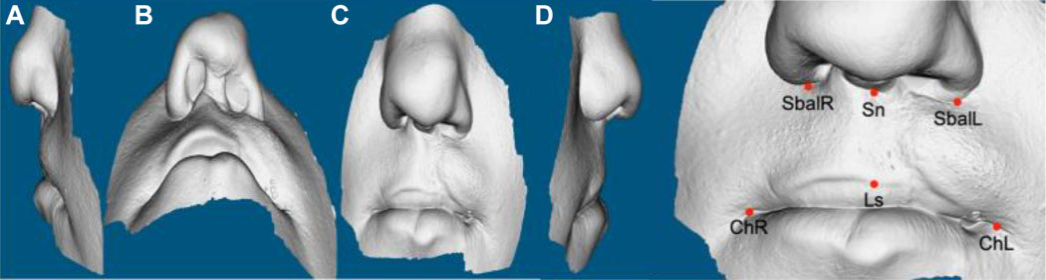
Nontextured 3-dimensional (3D) image of the nasolabial region captured by an intraoral scanner highlighting the landmarks used for the analysis.

Eighteen surgically managed, nonsyndromic UCLP cases were recruited, with the age range between 8 and 12 years. All cases followed the same surgical protocol for the management of UCLP, but not necessarily repaird by thesame surgeon. For each patient, only the nasolabial region was captured using an IOS (3Shape Trios 3rd generation A/S, Copenhagen, Denmark, 2011; Figure 1) to produce 3D image of the nasolabial morphology (Figure 2). The capture took 2 to 3 minutes, and this was carried out at the clinic, by the clinician, during routine appointments. The scanning sequence began at the left corner of the mouth, going through both the upper and lower lips, to the right corner of the mouth. The scanner was then rotated upward to capture the right side of the nose followed by the tip of the nose, then the left nostril. The imaging process was completed by recording the nasal bridge. These images were converted to stereolithography format to be viewed and processed in VRMesh software (VRMesh studio VirtualGrid). To avoid any influence of the color of the skin or the scar during the subjective evaluation process, the skin texture was removed from all images, which were viewed in gray scale with a standardized blue background (Figure 3).

### Objective Measurements of Upper Lip Scarring and Asymmetry

**Figure 4.**
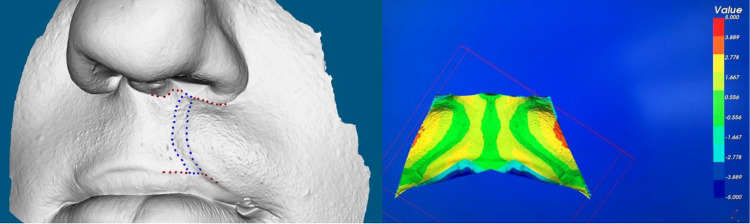
Quantification of the scar surface area (left) and asymmetry (right) of the upper lip.

Five landmarks were digitized on the 3D images to define the boundaries of the upper lip. The upper-lip-defining landmarks were the right alare, left alare, subnasale, right and left cheilion, and the vermilion border. To quantify the asymmetry, the upper lip regions were extracted from the 3D images according to their digitized anatomical boundaries. Mirror images of the defined regions were then obtained and superimposed on the original copy based on Procrustes surface registration method, and the disparity between the 2 images measured the asymmetry which was illustrated on color maps (Figure 4).

The scar surface area was identified manually on the 3D image. The tracing of the scar region began by marking the most prominent part of the vermilion border on each side of the scar and the most medial and lateral points of the scar at the nasal floor. This process and the remainder of the scar were outlined based on the surface topography using a digitally designated marker (Figure 4).

To assess the reproducibility of the measurements, the landmark digitization, tracing of the scar region, and the calculated measurements were repeated after one week by the same operator for all the captured images. The Wilcoxon signed-rank test was applied to assess the reproducibility of the landmark digitization and scar tracing. The association between the objectively measured surface area of the scar and asymmetry of the upper lip was tested using the Kendall rank correlation coefficient.

### Subjective Evaluation of Upper Lip Scarring and Asymmetry

Two assessment panels took part in the subjective evaluation process of the captured 3D images. The expert panel comprised of 5 clinicians who are familiar with the management of cleft lip and the second panel included 5 lay persons. All assessors underwent a mandatory caliberation exerciser before the start of the data analysis. A 5-point, Likert-type, ordinal scale was used to evaluate 5 different parameters (1) the degree of visibility of the scar length, (2) the continuity of the upper vermilion border, that is, scar width, (3) the disparity in the height of the cleft and noncleft sides of the upper lip, (4) the vertical asymmetry of the upper lip, and (5) the asymmetry of the upper lip in the depth or Z dimension (Supplemental Material).

Eighteen videos (one for each of the 3D image of the nasolabial region) using Auto Screen Recorder software. Each video was displayed once over a minute to allow sufficient time for evaluation. The videos displayed the 3D captured image in the following order: frontal view, right lateral profile, frontal view, left lateral profile, frontal view, submental view (worm’s eye), and ended with the frontal view to simulate the head movement during clinical examination.

The intrarater reproducibility was tested by repeating the evaluation after 6 months. The order of the patients was randomly changed in the second assessment session to avoid the impact of the severity of cases on the gradings. The Wilcoxon signed-rank test was applied to test the intraexaminer reproducibility. In addition, interrater reliability was investigated using the Kruskal-Wallis test to compare the mean scores of the professional and lay panels. Moreover, the Kendall rank correlation (T) was used to investigate the strength of the agreement between, and within, the two panel of assessors. The association between the subjective assessment and objective measurements of the residual asymmetry and lip scarring was investigated using the Kendall rank correlation. Data were analyzed using SPSS (IBM SPSS Statistics, version 24) and MINITAB software (Minitab, version 18.1).

## Results

The capture of the nasolabial region with IOS was readily achievable with minimal discomfort using a non-invasive method. The reproducibility of the landmark digitization was within 0.2 mm, and there was no statistically significant difference in the repeated measurement of the lip scar (*P* > .05). The repeated digitizations of the landmarks were strongly correlated (T = 0.9).

The Shapiro-Wilk test of normality demonstrated that the data did not follow the normal distribution curve and therefore nonparametric tests were applied for the statistical analyses.

Objective assessment of the 3D images showed that the lowest mean residual asymmetry was in the X-axis (0.425 mm) while the highest was in the Z-axis (0.699 mm). The correlation between the objectively measured scar surface area and asymmetry was weak (T < 0.2). Consistent moderate correlations (T > 0.45) were found between subjective and objective evaluations of the lip scarring, these were statistically significant at *P* < .05. The consensus between the subjective and objective assessments, however, was slightly lower regarding total asymmetry (T > 0.35). These findings were consistent among both assessment panels.

Moderate to strong correlations were found between the 2 assessment panels (T ranging between 0.5 and 0.9) with no significant difference (*P* > .05) regarding the mean score of the subjectively evaluated parameters (Table 1). On the other hand, intrarater reliability showed variable degrees of consistency (Table 2). The subjective assessment of the scar size was more reproducible than the assessment of lip asymmetry and lay assessors were more consistent than the professional panel. Albeit moderate to strong correlations (T ranging between 0.4 and 0.8), statistically significant differences were identified regarding the mean subjective grading of the asymmetry parameters which were scored by both the professionals and the laypersons (*P* < .05).

**Table 1.** Intraexaminer Reliability of the Subjective Evaluation.

Parameter	Professional panel		Lay panel	
	Wilcoxon signed-rank test (*P* value)	Kendall rank correlation	Wilcoxon signed-rank test (*P* value)	Kendall rank correlation
Scar length	.834	0.740^a^	.823	0.811^a^
Scar width	.075	0.768^a^	.972	0.482^a^
Vertical disparity at VB	.003	0.651^a^	.153	0.691^a^
Lip asymmetry (Y-direction)	.001	0.678^a^	.867	0.474^a^
Asymmetry (Z-direction)	.008	0.641^a^	.017	0.544^a^
Scar (length + width)	.390	0.820^a^	.753	0.789^a^
Total asymmetry	.000	0.718^a^	.055	0.726^a^
Total subjective score	.000	0.836^a^	.063	0.748^a^

Abbreviation: VB, vermilion border.

^a^ Correlation is significant at *P* < .01.

**Table 2.** Interexaminer Reliability of the Subjective Evaluation Between Professional and Lay Panels.

Occasion	Parameter	Kruskal- Wallis test (*P* value)	Kendall rank correlation
1	Scar length	.998	0.715^a^
	Scar width	.864	0.746^a^
	Vertical disparity at VB	.565	0.534^a^
	Lip asymmetry (Y-direction)	.264	0.609^a^
	Asymmetry (Z-direction)	.112	0.655^a^
	Scar (length + width)	1.000	0.793^a^
	Total asymmetry	.294	0.620^a^
	Total subjective score	.692	0.757^a^
2	Scar length	.998	0.602^a^
	Scar width	.864	0.568^a^
	Vertical disparity at VB	.565	0.602^a^
	Lip asymmetry (Y-direction)	.264	0.577^a^
	Asymmetry (Z-direction)	.112	0.615^a^
	Scar (length + width)	1.000	0.701^a^
	Total asymmetry	.294	0.625^a^
	Total subjective score	.692	0.682^a^

Abbreviation: VB, vermilion border.

^a^ Correlation is significant at *P* < .01.

## Discussion

This study demonstrated that IOS provides a reliable acquisition of 3D images of the nasolabial region, which facilitated both the subjective evaluation and quantification of the residual cleft deformity. In comparison to other nonionizing 3D facial imaging methods, IOS is easily portable, simple to use at the clinic and readily available in dental hospitals. The IOS is easy to use by the clinician, without the need for a trained photographer or a dedicated imaging room. The method provides high accuracy and fidelity of capturing 3D surfaces. The IOS proved accurate for extraoral applications and fabrication of maxillofacial prostheses (Liu et al., 2019; Ballo et al., 2019). There is no doubt that stereophotogrammetry provides the best imaging modality of the facial morphology of infants and young children due to the fast capture time, which is unmatched by any other technique. The major drawback of stereophotogrammetry though is the space which is mandatory for the multi-pod system, the need for a calibration process before capture, a trained photographer is need for image acquisition. In addition, the cost of the IOS is about 50% the cost of standard 2 pods stereophotogrammetry capture station.

Mosmuller et al. (2017) reported a weak correlation between asymmetry measurements on 2D and 3D images. Although both were objectively measured, they stated that this finding could be attributed to the fact that multiple perspectives of asymmetry of the nasolabial area was quantified on the 3D images compared to only the unidirectional contour of the nose and lip on the conventional photographs. Therefore, it is recommended and should be the standard routine to capture the nasolabial region using 3D imaging system.

In the present study, the statistically significant differences between the mean subjective scores of the asymmetry assessment within the professional panel could be attributed to the fact that they had varying level of experience with cleft management and therefore subjective inconsistency in interpreting labial asymmetry. Additionally, the washout period between the rating sessions in our study (6 months) was longer than that reported in previous studies which was limited to 1 to 2 months (Al-Omari et al., 2003, Mercan et al., 2018). The interpretation of the residual dysmorphology of surgically managed cleft cases is certainly challenging and there is a need for a tool to allow the objective analysis.

The assessment of the size of the scar is less complex and can be reliably evaluated subjectively. The findings of the present study support this concept by showing a higher level of reproducibility between and within the assessment panels. In addition, there was a stronger agreement between the subjective and objective evaluations regarding the lip scarring compared with the asymmetry assessment. One might argue that presenting the full face on colored 3D photographs for the qualitative assessment may have improved the correlation between the subjective and objective assessment of the nasolabial asymmetry. However, previous studies have contradicted this postulation and demonstrated that the surrounding facial features could influence the perception of the nasolabial appearance of surgically managed UCLP cases (Asher-McDade et al., 1991).

We acknowledge the small sample size regarding the subjective clinical evaluation that was limited to 6 assessors, but the moderate correlation that we detected between the objective measurements and the clinical evaluation of lip scarring was statistically significant. The results of the present study are consistent with previous reports on the correlation between subjective evaluation and objective measurement of cleft-related facial deformity (Meyer-Marcotty et al., 2010; Desmedt et al., 2015). Mercan et al. (2018) have carried out indirect anthropometric analysis on 3D images of the nasolabial region of UCLP cases. Seven cleft surgeons subjectively graded the same region, moderate to strong correlations were reported among the experts, who scored the quantified columellar angle, nostril width ratio, and lateral lip height ratio (Pearson correlation coefficient ranging from 0.34 to 0.8). However, the study was based on a limited set of linear and angular measurements, lip scarring, and asymmetry were not considered in the analysis. Similarly, the association between the subjective evaluation of the nasolabial appearance and the quantified nasolabial asymmetry on 3D images of UCLP cases was investigated (Desmedt et al., 2015). The regression analyses and reported *R* ^2^ values ranged from 0.11 to 0.25, which indicated that the aesthetic scores were poorly correlated with the measured nasolabial asymmetry.

The influence of facial asymmetry on the perception of facial appearance of UCLP cases has been investigated (Meyer-Marcotty and Stellzig-Eisenhauer et al., 2009), a moderate to strong correlations between midface asymmetry and the subjective evaluation by professional and lay assessors (Spearman correlation coefficient ranging from 0.615 to 0.757).

Clinically validated objective assessments are considered the gold standard for outcome measurements (Ayoub et al., 2011). However, the validity of the measurements should be tested against agreed clinical parameters to ensure the reproducibility of the clinical assessments. There is a considerable debate in the literature related to the most suitable group of assessors to conduct the subjective assessment of facial images due to the lack of agreement between surgeons, patients, and laypersons regarding the grading of residual dysmorphology (Trotman et al., 2007; Ayoub et al., 2011). Therefore, 2 panels of assessor were considered in this study. The IOS provided a reliable recording of lip scarring and the related asymmetry, which could be applied to assess the surgical outcomes of cleft repair and guide the decision-making process regarding the need for further corrective surgery. The IOS is a useful tool for the recording and the subsequently analyzing the nasolabial morphology, their availability in most of the clinics and dental hospitals should encourage multicenter studies. It will facilitate the development of outomated landmark recognition and machine learning to measure outcome for the improvemnt of surgical care.

## Conclusions

The use of the IOS provided an innovative and cost-effective application for capturing the complex 3D morphology of the nasolabial region. It is non-invasive and patient inconvenience during scanning is minimal. The analysis of the images captured by IOS has a satisfactory clinical validity for the objective quantification of residual dysmorphology which is valuable for improving the quality of the surgical repair of cleft lip and palate.

## Supplemental Material

Supplemental Material, Appendix_1 - The Validation of an Innovative Method for 3D Capture and Analysis of the Nasolabial Region in Cleft Cases Click here for additional data file. Supplemental Material, Appendix_1 for The Validation of an Innovative Method for 3D Capture and Analysis of the Nasolabial Region in Cleft Cases by Ashraf Ayoub, Adil Khan, Ali Aldhanhani, Hashim Alnaser, Kurt Naudi, Xiangyang Ju, Toby Gillgrass and Peter Mossey in The Cleft Palate-Craniofacial Journal
