# Supplementary material for: The Validation of an Innovative Method for 3D Capture and Analysis of the Nasolabial Region in Cleft Cases
Source: Cleft Palate Craniofac J. 2020 Aug 12;58(1):98–104. doi: 10.1177/1055665620946987 (PMC7739118; doi:10.1177/1055665620946987)
Supplement: Supplementary file 1 [file Appendix_1.docx]

Lip Scar and Asymmetry Assessment Form

Name of the Assessor:
Patient number:
Date of Assessment:

A. How do you assess the visibility of the scar length?

1. Non-Visible scar: No elevation or depression
2. Barely Visible scar: slight elevation or depression confined only to the upper vermilion border and not extending upward
3. Slightly Visible scar: slight elevation or depression extends up to 2-5 mm vertically above the upper vermilion border
4. Moderately visible scar: >5 mm extending upward
5. Very visible scar: very clear elevation or depression extends from the upper vermilion border to the nose (full length)

B. How do you assess the effect of the scarring on the Continuity (the gap) of the upper vermilion border of the upper lip?

1. No defect
2. Mild defect (vermilion notch): no loss of continuity
3. Moderate defect: step deformity
4. Severe defect: combination of step and gap deformity

C. How do you assess the difference in the height between the right and the left side of the upper lip?

1. No difference between right and left sides of the lip
2. Small difference (2mm) between the right and left sides of the lip
3. Moderate difference (2-5mm)
4. Severe difference more than 5mm but less than 10mm
5. Very Severe: the difference between the two sides is more than 10mm

D. How do you assess the vertical asymmetry of the upper lip?

1. Excellent symmetry: zero asymmetry
2. Satisfactoryy symmetry: up to 2mm asymmetry
3. Obvious asymmetry: 2-5mm Asymmetry
4. Poor symmetry: 5-10 Asymmetry
5. Severe asymmetry: more than 10mm asymmetry

E. How do you assess the asymmetry in the Z direction?

1. Grade I: zero asymmetry
2. Grade II: Mild asymmetry, up to 2mm
3. Grade III: Moderate asymmetry, 2-5mm
4. Grade IV: Severe asymmetry, 5-10mm
5. Grade V: very severe asymmetry: more than 10mm
